# Effect of a Specialized Nursing Subject Assignment on Perceived Knowledge and Skills to Care for Drug Users

**DOI:** 10.17533/udea.iee.v42n3e15

**Published:** 2024-11-02

**Authors:** Miguel A. Villegas-Pantoja, Mildred Astrid Moreno-Cruz, Martha Dalila Méndez-Ruiz, Carlos Reyes-Sánchez

**Affiliations:** 1 Nurse, PhD. Professor and Researcher. E-mail: mapantoja@docentes.uat.edu.mx. https://orcid.org/0000-0001-9917-8439 Universidad Autónoma de Tamaulipas Mexico mapantoja@docentes.uat.edu.mx; 2 Nurse, Masters. Professor. E-mail: mildred.moreno@docentes.uat.edu.mx. https://orcid.org/0000-0001-5295-8817 Universidad Autónoma de Tamaulipas Mexico mildred.moreno@docentes.uat.edu.mx; 3 Nurse, PhD. Professor and Researcher. E-mail: mdmendez@docentes.uat.edu.mx. Corresponding author. https://orcid.org/0000-0002-4527-0296 Universidad Autónoma de Tamaulipas Mexico mdmendez@docentes.uat.edu.mx; 4 Nurse, PhD. Professor and Researcher. E-mail: crsanchez@docentes.uat.edu.mx. https://orcid.org/0000-0001-7758-5906 Universidad Autónoma de Tamaulipas Mexico crsanchez@docentes.uat.edu.mx; 5 Faculty of Nursing at Nuevo Laredo, Universidad Autónoma de Tamaulipas, México. Universidad Autónoma de Tamaulipas Faculty of Nursing Universidad Autónoma de Tamaulipas Mexico

**Keywords:** nursing education research, substance-related disorders, knowledge, perception, investigación en educación de enfermería, trastornos relacionados con sustancias, conocimiento, percepción., pesquisa em educação em enfermagem, transtornos relacionados ao uso de substâncias, conhecimento, percepção

## Abstract

**Objective.:**

To identify the effect of a specialized subject on addictions on the knowledge and skills perceived by undergraduate nursing students to care for drug users.

**Methods.:**

This was a pre-experimental study of a single group with post- and pre-test measurements. The study included a sample of 59 students registered in the seventh semester in a public university in northeastern Mexico, who received 64 theoretical hours of the subject assignment “Nursing and addictions”, which was taught in face-to-face classes by professor nurses with training in the care of addictions. The emphasis was theoretical, where strategies, like clinical cases, screening instruments, and audiovisual material were used. Information was collected about sociodemographic data and the scale by Happel et al., of knowledge and perceived skills on the care of alcohol and other drug users was applied.

**Results.:**

The post-test registered higher scores in the subscales of overall knowledge on substance use (*W* = -4.532, *p* < 0.001), perceived knowledge on management of drug users (*W* = -5.909, *p* < 0.001), and perceived competences to care for alcohol and drug users (*t* = -8.000, *p* < 0.001).

**Conclusion.:**

The subject assignment analyzed contributed to increasing knowledge by nursing students regarding the phenomenon of addictions. As for perceived competences, although these increased significantly, they would have to be demonstrated in light of practical scenarios that confirm their persistence and impact on the quality of care.

## Resumen

**Objectivo.:**

Identificar el efecto de una asignatura sobre adicciones en los conocimientos y competencias percibidas por estudiantes de Licenciatura en Enfermería para el cuidado de usuarios de drogas.

**Métodos.:**

Estudio pre-experimental de un grupo único con mediciones *pre-test* y *post-test*. Se trabajó con una muestra de 59 alumnos matriculados en séptimo semestre de una universidad pública del noreste de México, quienes recibieron 64 horas teóricas de la asignatura “Enfermería ante las adicciones”, la cual fue impartida en clases presenciales por enfermeros docentes con formación en el cuidado de las adicciones. El énfasis fue teórico, donde se recurrió a estrategias como casos clínicos, instrumentos de tamizaje y material audiovisual. Se recolectó información sobre datos sociodemográficos y se aplicó la escala de conocimientos y competencias percibidas sobre el cuidado de usuarios de alcohol y otras drogas de Happel *et al*.

**Resultados.:**

En el *post-test* se registraron mayores puntuaciones en las subescalas de conocimientos generales sobre el uso de sustancias (*W* = -4.532, *p* < 0.001), conocimientos percibidos sobre el manejo de usuarios de drogas (*W* = -5.909, *p* < 0.001) y de competencias percibidas para cuidar usuarios de alcohol y drogas (*t* = -8.000, *p* < 0.001).

**Conclusión.:**

La asignatura analizada contribuyó a aumentar los conocimientos de los estudiantes de enfermería en torno al fenómeno de las adicciones. En cuanto a las competencias percibidas, aunque aumentaron significativamente, tendrían que demostrarse a la luz de escenarios prácticos que confirmen su persistencia e impacto en la calidad del cuidado.

## Resumo

**Objetivo.:**

Identificar o efeito de uma disciplina sobre dependências nos conhecimentos e habilidades percebidas por estudantes de Bacharelado em Enfermagem para cuidar de usuários de drogas.

**Métodos.:**

Estudo pré-experimental de grupo único com medidas pré-teste e pós-teste. Trabalhamos com uma amostra de 59 alunos matriculados no sétimo semestre de uma universidade pública do nordeste do México, que receberam 64 horas teóricas da disciplina “Enfermagem diante das dependências”, ministrada em aulas presenciais. ensinando enfermeiros com formação no cuidado de dependências. A ênfase foi teórica, onde foram utilizadas estratégias como casos clínicos, instrumentos de triagem e material audiovisual. Foram coletadas informações sobre dados sociodemográficos e aplicada a escala de conhecimentos e competências percebidas sobre o cuidado de usuários de álcool e outras drogas de Happel *et al.*

**Resultados.:**

No pós-teste, foram registradas pontuações mais altas nas subescalas conhecimento geral sobre o uso de substâncias (*W* = -4.532, *p*< 0.001), conhecimento percebido sobre o manejo de usuários de drogas (*W* = -5.909, *p*< 0.001) e percepção competências para cuidar de usuários de álcool e drogas (*t* = -8.000, *p*< 0.001).

**Conclusão.:**

A temática analisada contribuiu para ampliar o conhecimento dos estudantes de enfermagem sobre o fenômeno das dependências. Quanto às competências percebidas, embora tenham aumentado significativamente, teriam que ser demonstradas à luz de cenários práticos que confirmassem a sua persistência e impacto na qualidade dos cuidados.

## Introduction

Consumption of legal and illegal drugs poses a serious problem to public health, not only due to the high costs associated with their medical care, but also due to the numerous effects and consequences for the individual, the family, and the community.^1^ Las legal and illegal drugs, both under the denomination of psychoactive substances, are characterized for altering the central nervous system, causing disorders in the person's judgment function, behavior, mood, and a long list of related pathologies.^2^ Due to their effects on the cerebral structures involved in the perception of pleasure, are likely to create dependence (*i.e*., cause intense and persistent desire for the substance, as well as difficulties in controlling its consumption).^3^

Over the course of recent years, concern about drug consumption has increased, especially among young people. As evidence of this, almost two-thirds of premature deaths and one-third of the total burden of disease in adults are associated with diseases or behaviors that have their onset in youth, among them legal drug consumption.^4^ In fact, according to the World Health Organization, in 2020, over 1.5-million adolescents and young adults between 10 and 24 years of age died, equivalent to almost 5,000 deaths per day. Among the principal causes of death are injuries and traumas (including those caused by traffic accidents), violence, and self-harming behaviors. The foregoing demands actions aimed at preventing any type of addiction, as well as diminishing the consumption of psychoactive substances.^5^ In this sense, a line of action promoted to limit the spread of addictions is the training of health professionals in the area of ​​drug addiction, especially since their university formation.^6^^-^^8^ The case of nursing stands out, a discipline with a wide presence at all levels of the health system. However, when students graduate from the Nursing career, they often do not have sufficient knowledge about the drug phenomenon; an adverse situation for nursing students is that, as preventive agents, they also constitute a vulnerable population for experimentation and substance abuse, given their development stage and the context in which they perform. For example, a multicenter study carried out with nursing students^9^ determined that 36.1% consumed some type of illegal drug, more commonly marihuana (35.9%), ecstasy (6.9%), and cocaine (5.8%). Not less relevant is the consumption of legal drugs; regarding tobacco, a study conducted in Spain ^(^^10^ determined overall prevalence around 29.7%. Furthermore, it is estimated that 61.0% of nursing students in a study conducted in the United States ^(^^11^ have recurred to episodic excessive alcohol consumption (that is, drinking five or more alcoholic beverages per occasion). 

Added to the high prevalence mentioned, students may have inconsistent knowledge about drugs. For example, in a research with students from the Nursing program at Universidad Autónoma de Coahuila, Mexico,^12^ 70.9% of the participants had a low level of knowledge about caring for patients who consume alcohol and tobacco. In another research in Peru, students described the risks of consuming alcohol and tobacco as quite dangerous, but classified marihuana consumption as slight health risk.^13^ Situations, like the aforementioned, evidence broad areas of opportunity in terms of education on the phenomenon of addictions among nursing students. Unfortunately, at least within the context of Mexico, many higher education institutions that have a Nursing program do not include specific subject assignments or sufficient program content that permit students to care for patients with drug addictions. This is so, despite efforts aimed at establishing specific nursing skills to reduce drug demand.^14^^,^^15^ Thereby, although it is important for education authorities to include assignments to acquire the knowledge and skills required for prevention and caring for drug users, it is also a priority for professors and researchers to evaluate the educational benefits offered by said subjects; this is because it is rarely determined how capable students are based on programs implemented. 

Under that perspective, this study represents an effort to explore student learning in an undergraduate Nursing program located in northeastern Mexico, who were registered in a specific subject on addictions, of recent implementation and with a duration of 16 weeks. Although prior local research has been carried out ^(^^16^ on the personal benefits this subject assignment could bring to students (in terms of changes in their beliefs and intentions to consume), what is still unknown are the cognitive characteristics with which students end up (which are related to improved quality of patient care). Due to the foregoing, the aim of this study was to identify the effect of a specialized subject assignment on addictions on the knowledge and skills perceived by undergraduate nursing students to care for drug users.

## Methods

This research had a single-group pre-experimental design^17^ with pre- and post-test measurements. The group subjected to the educational intervention included 59 university students from three classrooms that received the subject assignment “Nursing and addictions”. The inclusion criteria were to be a student formally enrolled in the regular period of the undergraduate Nursing program. Given that all the classrooms from the same academic period took the subject assignment, no equivalent comparison group could be found. The participants were in their daily classroom environment (classrooms with face-to-face modality), with the condition of not missing more than three sessions during the entire period (the average was 0.40 absences, SD = 0.68). The study was conducted during the first half of 2022 (third cohort since the subject was included in the nursing curriculum, during the second half of 2019). The sample size was sufficient to detect an effect size from *d* = 0.4, considering an alpha error of 0.05 and power of 83.8% to perform repeated measurements analysis under a non-parametric test (for parametric tests of repeated measurements the power was 85.5%).

The undergraduate Nursing program is conformed of 10 semesters, with the last two corresponding to a type of internship (denominated social service in Mexico). The subject assignment is offered as an elective during the seventh semester (that is, education authorities can substitute it for any other subject in certain academic periods). The class is made up of 64 hours, administered four hours per week. Three professors with previous experience in teaching the subject participated: one had a master's degree and two had PhD degrees in nursing sciences; in all cases, they had trajectories in research on addictions. At the time of the study, it was a subject with a theoretical emphasis because it was not accompanied by mandatory hours of specialized practice outside the classroom (partly due to the lack of specialized areas of care for drug addictions). The subject addresses the neuropsychological theoretical foundations underlying the development of addictions, the symptomatology, diagnosis, and treatment of legal and illegal substances, as well as nursing care. Teaching and learning strategies were used, such as clinical cases, application of screening instruments, and emergency care videos. The pre-test measurement was made prior to the first session and the post-test measurement was carried out after completing the period of classes (but shortly before the final ordinary evaluation of the subject assignment).

The instruments used for the measurements were: (i) a data sheet consisting of 17 questions aimed at identifying sex, age, experience in caring for alcohol or drug users, and the prevalence of personal use of substances (alcohol, tobacco, marihuana, heroin, cocaine, amphetamines/methamphetamines and controlled medications); and the prevalence measurements included consumption of the aforementioned psychoactive substances at some point in life, that is, the global prevalence; and (ii) the Scale on perceived knowledge and skills regarding the care of drug users developed by Happel *et al*.,^18^ aimed originally at knowing the needs and areas of opportunity of health professionals to work with alcohol and drug users. 

For the purposes of this research, a prior pilot test was carried out on a sample of 25 nursing students, where understanding and reliability were assessed. The questions in this instrument belong to four areas considered relevant among the staff caring for drug users: (i) general knowledge on substance use, (ii) frequency of recording substance use history; (iii) perceived knowledge on management of drug users; and (iv) perceived competences to care for drug users. Of the four areas mentioned, the items of the second one, aimed at knowing the occasions in which the student takes a record of the consumption of substances in drug-addicted patients, were not applied. The foregoing is based on the fact that a considerable proportion of the sample had never practiced caring for patients with drug addiction, so the post-test measurements would be a biased. 

With respect to the area of overall knowledge on substance use, it is composed of 17 multiple-option items (11 items of three options and six of two options). These questions address mainly knowledge on drug generalities, treatments, and symptoms in substance users. In all the questions, only one of the options is considered correct (coded dichotomously with the value 1 as the reference for successes), so that the score ranges between 0 and 17 points (higher scores mean greater knowledge). Because there was no established cut-off point, any score above the absolute mean of this subscale (8.5 points) was considered an indicator of the minimum acceptable knowledge threshold.

The subscale of perceived knowledge on management of drug users includes eight Likert-type items with four options (from *Much knowledge = 3* to *No knowledge = 0*). The score ranges between 0 and 24 points; a higher score suggests greater perception of knowledge about nursing care in addictions. In this research it was identified that it has an adequate Cronbach's alpha coefficient of 0.848. Finally, the competency subscale for caring for drug users has 10 Likert-type items with four options (from *Very competent = 3* to *Not competent = 0*); the score ranges from 0 to 30 points. A higher score indicates that the participant feels more competent or skilled in working with users of alcohol or other drugs. The reliability of this subscale is satisfactory (( = 0.934).

Regarding the data collection procedures, the first step consisted in securing authorizations from the institutional research and ethics committees. Once this was achieved, the necessary arrangements were made with the educational authorities. The researchers personally invited the students to their respective classrooms (just before their classes began); orally explained the objective of the study and collected written informed consents. At this point, 68 students accepted to participate, and applied the pre-test measurement. Administration of the instruments took just over 15 minutes. From that moment on, the students were allowed to continue their classes and activities as normal. Precisely at the end of the subject (16 weeks later), but before the regular or final evaluation, the post-test measurement was taken. At the end of the study there was a loss of nine participants (that is, an attrition of 13.23%). Said cases corresponded to students who abandoned or postponed their studies and their exclusion determined the final sample size. 

The analyses included descriptive and inferential statistics. Regarding the descriptive approach, measures of centrality, dispersion, as well as frequencies and relative frequencies (%) were used. The inferential approach focused on the comparison of repeated measurements. Thus, using the Kolmogorov-Smirnov test with Lilliefors correction, it was decided that for numerical variables that had a normal distribution (*p* > 0.05) the *t* test for related groups was appropriate. On the other hand, the Wilcoxon test was used when a normal distribution was not presented (*p* < 0.05). The McNemar test was used to compare categorical variables between repeated measurements. For comparisons between two independent groups, the non-parametric Mann-Whitney U test was used. Analyses were performed in SPSS version 24.0 (for the OSX operating system).

This study complied with national ethical dispositions (regulation of the general health law on research in Mexico) and international ones in force (Declaration of Helsinki). It was characterized by being anonymous, confidential and voluntary, where informed consent was required and signed by the participants. It was also approved by the institutional Research and Ethics Committee (registration CA-A017-2018).

## Results

Regarding the descriptive data of the participants, the majority were women (83.1%, ƒ = 49) with a mean age of 21.54±1.02 years. At the beginning of the study, more than half reported not having had any experience caring for alcohol or drug users (55.9%, ƒ = 33). The overall prevalence of substance use among students was as follows: alcohol 89.8% (ƒ = 53), tobacco 50.8% (ƒ = 30), marihuana 33.9% (ƒ = 20), controlled drugs 5.1% (ƒ = 3), cocaine 3.4% (ƒ = 2), and amphetamines 1.7% (ƒ = 1). 

On the general objective of the study (identifying the effect of a subject assignment on addictions on the perceived knowledge and skills to care for drug users), Table 1 shows that significant changes were noted at the end of the research. It is noteworthy that towards the post-test measurement, higher raw scores were recorded for general knowledge on substance use, perceived knowledge on management of drug users, and perceived competences to care for alcohol and drug users. That is, all three indicators improved after completing the assignment program. 

**Table 1:** Differences between perceived knowledge and skills to care for drug users upon completing the study

Variables	Pre-test			Post-test		Test statistic
*M*± *SD*	*95% CI M*	*M*± *SD*	*95% CI M*	
Overall knowledge on substance use	8.71±1.70	8.26-9.15	10.15±1.85	9.66-10.63	*W* = -4.532^*^
Perceived knowledge on management of drug users	8.83±3.97	7.79-9.86	14.27±4.00	13.22-15.31	*W* = -5.909^*^
Perceived competences to care for drug users	12.37±5.94	10.82-13.92	19.06±4.93	17.78-20.35	*t* = -8.000^*^

*Note*: *t* = *t* test for two related groups; 95% CI M = confidence intervals at 95% around the mean; *W* = Wilcoxon’s test; ^*^ *p* < 0.001

Specifically, in the area of general knowledge on substance use, upon analyzing its items, of the eight with significant changes (Table 2), five were about knowledge related to alcohol use. In fact, in this subscale it is estimated that on the moment of the pre-test 57.6% (ƒ = 34) of the participants exceeded the minimum threshold of 8.5 points, a situation that increased to 84.7% (ƒ = 50) on the moment of the post-test. In percentage terms, the pre-test moment registered 50.01% correct responses, while increasing in the post-test to 56.99%

**Table 2:** Some items from the area of overall knowledge on substance use registering significant changes

Items	Pre-test %	Post-test %	Test statistic
2. Maximum recommended amount of alcohol that men can consume in one day	20.3	69.5	*(*(^2^ = 27.129^***^
3. Maximum recommended amount of alcohol that non-pregnant women can consume in one day	62.7	78.0	*(*(^2^ = 4.764^*^
5. Vitamin that patients with alcohol dependence should take to avoid memory problems and confusion	27.1	66.1	*(*(^2^ = 19.592^***^
10. Warning sign warns of possible heroin overdose in sleepy patients with erratic behavior	39.0	61.0	*(*(^2^ = 7.347^**^
11. Upon suspicion of heroin overdose in a nonreactive patient, what medication should be administered immediately?	32.2	66.1	*(*(^2^ = 12.500^***^
12. The diagnosis of dependence on a substance is characterized by the need for a marked increase in the amount of the drug to achieve the effect desired by the user.	69.5	88.1	*(*(^2^ = 6.368^*^
15. The use of a benzodiazepine is appropriate to manage alcohol withdrawal symptoms.	40.7	62.7	*(*(^2^ = 5.827^*^
17. Many symptoms associated with alcohol withdrawal can be adequately managed without medication.	47.5	78.0	*(*(^2^ = 11.571^***^

*Note:* Percentages refer to the number of right answers in each item; *(*(^2^ = McNemar test statistic; ^*^ *p* < 0.05; ^**^ *p* < 0.01; ^***^ *p* < 0.001

**Figures 1A and 1B:**
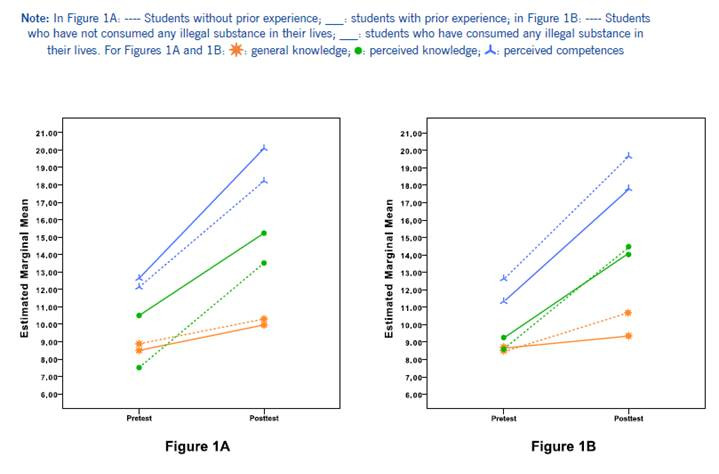
Study indicators in function of: **A-** Prior experience in caring for alcohol and drugs user, and **B-** Consumption of any illegal drug

To end, given that 55.9% of the participants reported having had some type of experience of caring for drug users, and 35.6% of them consumed some illicit substance in their lifetime, it was analyzed if said attributes constituted confounding variables. In this sense, it was identified that those with prior care experience, began the study with more perceived knowledge (*U* = 242.00, *p* = 0.004), although they did not have more general knowledge (*U* = 382.00, *p* = 0.465) or perceived competences (*U* = 394.50, *p* = 0.598). Figure 1A illustrates how the mean of perceived knowledge on management of drug users during the pre-test was higher among those with prior care experience (*M* = 10.50, *Med* = 11.0) compared with those who did not have such experience (*M* = 7.51, *Med* = 9.0). However, it is noteworthy that this attribute did not seem to be a determining factor in the fact that on completion of the study an increase in any of the three indicators of interest was recorded, given that the intergroup comparisons were not significant (*p* > 0.05). This can be seen from the relative closeness and parallelism of the scores of both groups of students in the post-test.

In the case of those who consumed any illegal substance (Figure 1B), they began the study with similar indicators compared to their non-consumer peers: perceived knowledge (*U* = 352.50, *p* = 0.459), general knowledge (*U* = 398.50, *p* = 0.994), perceived competences (*U* = 350.00, *p* = 0.437). Nonetheless, upon analyzing the measurements during the post-test, significant differences were found in general knowledge scores (*U* = 248.50, *p* = 0.015). It can be noted that the score was higher among non-consumers of any illegal substances during their lifetime (*M* = 10.55, *Med* = 11.0) compared to those who consumed them (*M* = 9.42, *Med* = 9.0). Although a causal direction cannot be determined, this suggests that consumption of any illicit substance is an attribute to consider when interpreting the analyses.

## Discussion

Through this study, it was identified that participants who received the assignment on nursing care for drug users experienced significant improvement in terms of general and perceived knowledge on substance use, as well as in the skills perceived to care for alcohol and drug users. With respect to overall knowledge acquired by the participants, improvements identified are in line with the literature that points out the benefits of formal subject assignments on addictions in terms of filling the knowledge gaps of nursing professionals.^19^ Nevertheless, an outstanding aspect is that - at least from the theoretical point of view - the knowledge gained by the students places them at levels comparable to those reported in studies carried out in countries, such as Australia. For example, Happel *et al.,*^18^ conducted a survey among health professionals to verify their knowledge regarding caring for users of alcohol and other substances. Their sample was comprised by 79.89% nursing professionals with > 10 years of clinical experience (71.7%). Using the same instrument as our study, they determined that, averaging all the items, there was 61.88% of correct answers; not distant from the 56.99% with which the students in the present study ended. Something similar was reported by Carta *et al*.,^20^ who implemented a two-day educational workshop to increase knowledge and attitudes with respect to alcohol and drug use. Said study recruited a sample of 378 professionals specialized in mental health; of which 77.5% were nursing staff and > 60.0% had over 10 years of professional experience. The right answers obtained in the pre-test measurement were 60.45% with the same instrument. 

As observed, in the Australian research, most of the nursing staff had professional experience and even greater academic formation (not leaving aside that these would be professionals trained in a country with high levels of development). These could be aspects that prevent a fair comparison with our data, but which put the percentages achieved by the students into context, given that the majority of our students have never provided care in addictions and had less clinical experience at the moment of the study (consider that the clinical experience could include training opportunities on addictions, care for drug addiction users, or of interaction with other trained professionals). It will be important to determine if knowledge is implemented directly during their professional practice, besides persisting over time. Currently, no measurements are available to permit corroborating these aspects. 

With regards to scores on perceived knowledge and skills to care for drug users, although often not listed as the main objective of the subject assignments, psychological theories suggest that perceptions could be relevant as precursors of behaviors.^21^ Therefore, these should also be of interest for academia. In this case, if we wish to promote adequate care for drug users (this is a behavior), it is also useful - and probably necessary - to elevate the set of determinants proximal to said behaviors, such as perceptions, beliefs, and intentions. In this sense, given that individuals tend to interpret information selectively according with their beliefs and prior experiences,^21^^,^^22^ the subject assignments constitute opportunities to influence on said constructs through information and activities carried out. Other studies^16^^,^^23^ have demonstrated that this assignment permits modifying personal beliefs with respect to drugs, so it is expected that this will translate into the execution of desirable behaviors in the future. However, the lack of clinical practice associated with this subject remains as a major pending issue, which could provide experiences that facilitate behavioral changes through improving perceived behavioral control.

Following the same line of theories that explain the determinants of behaviors,^21^^,^^22^ the effect that previous substance use by students could have is discussed (which in this study was high because 35.6% had used some illicit substance at some point in their lives). Although at the beginning of the study the consuming and non-consuming students started on equal terms, at the time of the post-test there were differences in favor of the non-consumers in terms of the overall knowledge acquired. Even if it is not possible to determine whether consumption of illegal substances is the determinant of poor performance, it is important to consider that the socioeconomic, emotional, and physiological context of users is often adverse, which could have an impact on learning processes. Likewise, the literature is clear that health professionals who are active users (or who have positive attitudes towards drugs) may be less efficient agents,^24^ given the repercussions of substance consumption on beliefs, attitudes, and motivations. Thus, although measuring and addressing student involvement with substances is beyond traditional academic purposes, it may be necessary for nursing educators to direct care actions toward students. According to the theoretical postulates mentioned, said actions could indirectly improve the quality of care provided by graduates.

It is important to highlight that diverse limitations exist in relation to this study. Inherent to the study design, by not having a control group, it is not possible to determine whether the changes are totally attributable to the subject assignment and not to other uncontrolled factors (such as age, gender, or beliefs). Also, because there was no random selection and the study was located in a very specific geographic context, it is difficult to generalize the findings to other populations or educational contexts. In fact, the city where the study took place has been characterized by the continuous presence of illegal substance trafficking, so that the local population could have particularities (cultural and psychological) that would not be seen in other areas of the country. It would also be appropriate to carry out medium-term follow-ups, since the study only measured the immediate effects of the subject assignment. In that sense, knowledge can diminish over time, especially if not put into practice. In addition, some threats to internal validity include relying on self-reporting instruments and a lack of control over possible changes that may occur in participants throughout the study (for example, learning by means other than through the subject assignment). It is recommended to take these limitations into account when designing future research to fully understand the effects of the subject assignment.
